# Randomised, double-blind, parallel group, placebo-controlled, trial of Bactek for the prevention of lower respiratory tract infections in preterm infants in the UK: BALLOON study – study protocol

**DOI:** 10.1136/bmjopen-2025-107929

**Published:** 2026-03-09

**Authors:** Sarah Joanne Kotecha, John Lowe, David Gillespie, Mahuampi Perez-Alijas, Ali F Aboklaish, Tarirai Lincoln Mahachi, Oliver Sebastian Cumming, Debbie Harris, Marie Hubbard, Emma Thomas-Jones, Tim Jones, Kirstin Ladell, Catherine Moore, Ian Humpreys, Jonathan Grigg, Janet Berrington, Sailesh Kotecha

**Affiliations:** 1 Department of Child Health, Cardiff University, Cardiff, UK; 2 Centre for Trials Research, University of Cardiff, Cardiff, UK; 3 Department of Child Health, Cardiff University School of Medicine, Cardiff, UK; 4 Neonatal Unit, University Hospitals of Leicester NHS Trust, Leicester, UK; 5 University Hospital of Wales, Cardiff, Wales, UK; 6 Cardiff University, Cardiff, UK; 7 Paediatric Respiratory, Queen Mary University London, London, UK; 8 Newcastle Upon Tyne Hospitals NHS Foundation Trust, Newcastle upon Tyne, UK

**Keywords:** NEONATOLOGY, Randomized Controlled Trial, PAEDIATRICS

## Abstract

**Introduction:**

A significant proportion of infants born at ≤29+6 weeks’ gestation develop lung disease during the neonatal period, thus putting them at risk of developing prematurity-associated lung disease in childhood and adulthood. After discharge from the neonatal unit, pre-existing lung disease in preterm-born infants is exacerbated by (often frequent) respiratory viral infections requiring greater health utilisation, including hospital admissions, than their term-born equivalents. Opportunities to prevent viral infections in infancy are largely limited to anti-respiratory syncytial virus (RSV) antibody prophylaxis and recently maternal RSV immunisation, but in term-born infants, trained immunity-based vaccines such as Bactek (MV130, Inmunotek, Spain) are increasingly used. Bactek provides a promising therapeutic avenue for preterm-born infants to target postdischarge respiratory viral infection in this vulnerable group of infants. The BALLOON study aims to assess this treatment in a very/extremely preterm-born population and determine if treatment with the trained immunity-based vaccine Bactek decreases the risk of unscheduled visits to healthcare professionals for lower respiratory tract infections, when compared with placebo. Included infants are born at ≤29+6 weeks’ gestation and treated daily from term-equivalent (37–43 weeks’ postmenstrual age, PMA) or from discharge, if earlier, up to 1 year of corrected age.

**Methods and analysis:**

542 infants are being recruited prior to discharge by neonatal units in the UK. They are being randomised to receive Bactek or placebo, once daily dose of 2 sprays (each 0.1 mL) of IMP (300 Formazin Turbidity Units), from 37 to 43 weeks’ PMA or discharge if earlier up to 1 year of corrected age. The primary objective is to assess if sublingual Bactek spray decreases the risk of health professional diagnosed lower respiratory tract infections (LRTIs) (unscheduled visits to general practitioners, accident and emergency departments and hospital admissions) between enrolment and 1 year of corrected age. Secondary outcomes include the number of parent-reported, health professional-confirmed unscheduled visits for LRTIs, the time to first parent-reported, health professional-confirmed unscheduled visit for LRTI, parent-reported wheeze episodes (identification aided by WheezeScan (Omron, Japan)), parent-reported use of respiratory medications, growth (weight, length and head circumference), parent(s)/guardian(s) reported time missed from work and/or nursery time missed for the infant and volume of adverse reactions. Viruses associated with LRTIs will also be identified.

**Ethics and dissemination:**

Ethics permission has been granted by the Wales Research Ethics Committee 3 (Ref 24/WA/0181), and regulatory permission by the Medicines and Healthcare Products Regulatory Agency (CTA reference 21323/0063/001-0004). The study is registered on ISRCTN (ISRCTN14019493). Findings will be disseminated via national and international peer-reviewed journals, and conferences. Oversight of the study is being provided by an Independent Data Monitoring Committee and an independent Trial Steering Committee (TSC). The Trial Management Group (TMG) meets every month.

**Trial registration number:**

ISRCTN14019493.

**STRENGTHS AND LIMITATIONS OF THIS STUDY**

- Randomised, double-blind, parallel group, placebo-controlled, trial design to enhance reliability of results using predetermined primary outcomes.
- Use of computer-generated randomisation and allocation concealment will avoid risk of selection and allocation bias.
- All potential lower respiratory tract infections will be verified by clinical staff and will be differentiated from other causes of wheezing, including bronchopulmonary dysplasia.

## Introduction

 A significant proportion of infants born at ≤29+6 weeks’ gestation develop lung disease during the neonatal period, thus putting them at a significant risk of developing prematurity-associated lung disease in childhood and adulthood,^1^ including premature development of chronic obstructive pulmonary disease. In a recent systematic review, we reported that infants with bronchopulmonary dysplasia (BPD) have a ~17% decrease in percent predicted forced expired volume in 1 s in later life.^2^ At discharge from the neonatal unit, any pre-existing lung disease is exacerbated by frequent respiratory viral infections requiring greater health utilisation, including hospital admissions, than their term-born equivalents. For example, Drysdale reported that 73 of 159 (46%) discharged infants with median gestation of 34 (range: 23–36) weeks developed lower respiratory tract infections (LRTIs)^3^ during the first year of life. Importantly, those who had viral infections had worse lung function at 1 year of corrected age when compared with those who did not.^4^ Rates of LRTIs are higher in preterm-born children in the first year of life compared with term-born children.^5^ More recent UK data using record linkages reported that 1666 preterm infants born at <32 weeks’ gestation followed up to 5 years of age had adjusted incidence rate ratios for LRTIs of 2.79 (2.59, 3.01) when compared with 253 277 term-born controls.^6^ Even in term-born infants, evidence, after such viral infections, points towards the development of preschool wheezing, but in preterm-born infants, the consequences are greater in preterm-born infants due to added insults to any pre-existing lung disease.

Opportunities to prevent viral infections in infancy for preterm infants are currently largely limited to anti-respiratory syncytial virus (RSV) antibody prophylaxis and more recently maternal RSV immunisation during pregnancy, but in term-born infants, trained immunity-based vaccines (TIBVs) such as Bactek (MV130, Inmunotek, Spain) are increasingly being used in infants at risk of developing wheezing.^7 8^ Bactek, a sublingual spray, is a polybacterial preparation of heat-inactivated *Staphylococcus aureus, Staphylococcus epidermidis, Streptococcus pneumoniae, Haemophilus influenzae, Klebsiella pneumoniae and Moraxella catarrhalis*, which can boost innate immunity and enhance T-cell responses.^9^,^11^ Bactek is not a conventional vaccine but is generally regarded as a TIBV, working by generating broad heterologous protection against pathogens related and unrelated to its formulation.^12 13^ By exerting actions via activation of both the innate and adaptive immune systems, both short and long-term memory is generated.^14^ Martín-Cruz *et al* have recently discussed the mode of action of TIBVs in detail.^14^

There is evidence for the efficacy of Bactek, and other TIBVs, in studies of infants and young children in decreasing wheezing and respiratory exacerbations (an acute increase in the severity of respiratory symptoms).^8^ These studies have often included a small number of preterm-born infants/children. Systematic reviews of studies using bacterial lysates have confirmed decreased wheezing in preschool children (mean difference of −2.35, 95% CI −3.03 to –1.67), reduced duration of each wheezing episode and absence of adverse reactions for pooled studies of Bactek and the bacterial lysate OM-85.^15^ Moreover, rates of LRTIs are also decreased; for example, Liao and Zhang reported the results of a clinical trial in the treatment of asthma combined with recurrent RTI in children, concluding that OM-85 reduces acute RTIs.^16^ A further trial studying OM-85 in infants at risk of asthma reported OM-85 also decreased the incidence of severe lower RTI in early life.^17^ A recent study in adults and children reported a significant decrease in the median number of antibiotic courses and concluded that Bactek is an effective strategy for the prevention of respiratory infections.^7^ Thus, there is growing proof of concept that Bactek provides a promising therapeutic avenue.

The current published data are likely to be applicable to the preterm-born population given their vulnerability to respiratory disease. However, there is supporting evidence including Nieto’s Bactek study^8^ reporting fewer wheezing attacks (3.0 vs 5.0) in the whole cohort (from which 16.7% were preterm-born children) at a median age of 24 months. Since it is unclear if the effects are conclusive in an exclusively preterm-born population, a trial focussing only on very/extremely preterm-born infants who are discharged when they reach their due date is required. An additional ongoing trial is investigating OM-85 in infants born after 30–36 weeks of gestation, which will broaden the evidence base across the spectrum of prematurity.^18^

In summary, preterm infants born ≤29+6 weeks’ gestation are at increased risk of additional damage to their already impaired lungs through LRTIs over their first year of life. Data from studies using TIBV report reductions in wheeze/asthma in other related populations which include preterm-born infants, a small number of whom were less than 1 year of age. The outcomes for the preterm-born population have not been reported separately. Therefore, the BALLOON study is assessing whether treatment with the TIBV Bactek decreases the risk of unscheduled visits to healthcare professionals for LRTIs.

## Methods and analysis

### Primary objective

The primary objective of the BALLOON trial is to investigate if sublingual Bactek spray, when compared with placebo, decreases the risk of health professional diagnosed LRTIs (after unscheduled visits to general practitioners (GPs), accident and emergency departments (A&Es) and hospital admissions) between term-equivalent 37–43 weeks’ postmenstrual age (PMA) or discharge if earlier and 1 year of corrected age in preterm infants born ≤29+6 weeks’ gestation.

### Design

BALLOON is a multicentre, double-blind, randomised, two-stage, placebo-controlled trial.

A subset of sites has been selected to collect blood samples at baseline, 6 months and 1-year corrected age. Collection of these blood samples for use in future research on trained immunity and related areas is an optional aspect of parental/guardian consent at these sites. A subset of sites has also been selected to perform lung function measurements (impulse oscillometry) using the Tremoflo N-100 machine (Thorasys, Montreal, Canada) at baseline, 6 months and 1-year corrected age. Collection of these measurements is an optional aspect of parental/guardian consent at these sites. The objectives of the substudies are translational in nature and outside the scope of the main trial protocol.

### Setting

Infants are being enrolled from UK tertiary neonatal intensive care units. Infants are identified by the local site study team prior to discharge from the neonatal unit and screened against the inclusion/exclusion criteria.

### Inclusion criteria

1. Birth at gestational age ≤29+6 weeks (including infants born as one of a multiple birth).
2. In the opinion of the principal investigator (PI) at the recruiting site, follow-up is likely to be feasible (ie, routine outpatient appointments will be at the recruiting site, locality of infant’s residence so follow-up to 1-year corrected age is possible.)
3. Survival to 1-year corrected age is anticipated.
4. In addition to infants without an eventful course, the following groups are eligible for inclusion:
  - Infants diagnosed with BPD
  - Infants discharged home on oxygen
  - Infants who have had necrotising enterocolitis
  - Infants on oral or nasogastric tube feeding
  - Infants with treated patent ductus arteriosus
  - Infants with neurological disorders
  - Infants with resolved sepsis

### Exclusion criteria

1. Presence of major surgical, cardiac or congenital abnormalities (not including patent ductus arteriosus or patent foramen ovale).
2. Contraindication of Bactek as specified in the investigator’s Brochure.
3. Participation in other interventional trial that precludes participation in BALLOON.
4. Primary immune deficiencies.

### Trial intervention

Bactek or placebo to match is packaged, labelled and QP released by Inmunotek (Madrid, Spain). St Mary’s Pharmaceutical Unit (SMPU, Cardiff and Vale University Health Board) (MIA (IMP) 359929) imports the IMP to the UK and provides final regulatory release. SMPU warehouses the IMP supply and distributes it to the study sites.

The dosing schedule of 2 sprays (each 0.1 mL) of IMP (300 Formazin Turbidity Units (FTU)) is being administered, once daily, from 37 to 43 weeks’ PMA or discharge if earlier up to 1 year of corrected age. A video showing how to administer the IMP is shown on the BALLOON website. All data thus far collected, including infants, preschool aged children and adults, support using the same dose of 2 daily sprays of 300 FTU.^812 19^,^21^

### Blinding

Bactek and placebo are packaged as blinded pairs of sprays, which in combination provide sufficient supply for at least 3 months of treatment. Five boxed pairs of sprays are presented in a ‘treatment pack’, sufficient to cover the treatment period. Labelling was performed by Inmunotek. The randomisation list was provided by the Centre for Trials Research (CTR), Cardiff University.

### Active arm

The active arm comprises Bactek (Inmunotek) a sublingual spray comprising a polybacterial preparation of heat-inactivated *S. aureus* (15%), *S. epidermidis* (15%), *S. pneumoniae* (60%), *H. influenzae* (3%), *K. pneumoniae* (4%) and *M. catarrhalis* (3%).

### Control arm

The placebo arm is a sublingual spray comprising all excipients of the active IMP, but no inactivated bacteria.

### Outcomes

#### Primary outcomes

The primary outcome is the presence/absence of parent-reported, health professional-confirmed unscheduled visits for LRTIs to GPs, A&Es, paediatric assessment units or hospital admissions between 37 and 43 weeks’ PMA (or discharge if earlier) and 1-year corrected age, compared between the two treatment arms.

An LRTI is defined as:

- Fever (≥38°C) or a runny nose (rhinitis) for at least 12 hours, plus one additional symptom from the following:
  - moist cough
  - wheezing
  - shortness of breath
  - tachypnoea
  - intercostal recession
  - poor feeding

Secondary outcomes include:

1. The actual number of parent-reported, health professional-confirmed unscheduled visits for LRTIs to GPs, A&Es, paediatric assessment units and hospital admissions between 37 weeks’ PMA (or discharge if earlier) and 1-year corrected age. The LRTI will be considered to be a new episode if there is an intervening period of at least 72 hours between episodes when the infant is well.
2. The time to first parent-reported, health professional-confirmed unscheduled visit for LRTI to GPs, A&Es, paediatric assessment units and hospital admissions between 37 and 43 weeks’ PMA (or discharge if earlier) and 1-year corrected age.
3. Parent-reported wheeze episode between 37 and 43 weeks’ PMA or discharge if earlier and 1-year corrected age.
  A wheeze episode is defined as an episode of wheezing that lasts at least 1 day with signs of increased work of breathing, such as shortness of breath, cough or chest retraction or with any combination of these additional symptoms. A WheezeScan device is provided to parent(s)/guardian(s) to detect and differentiate wheezing from other breath sounds.
4. Identification of virus(es) associated with LRTIs.
5. Parent-reported use of respiratory medications including bronchodilators, antibiotics and systemic corticosteroids.
6. Somatic growth differences (weight, length and head circumference).
7. Parent(s)/guardian(s) reported time missed from work and/or nursery time missed for the infant.
8. Volume of adverse reactions.

### Patient and public involvement

The study has had significant input from two patient and public involvement (PPI) coapplicants starting from the development of the grant, who have both lived through the experiences of the neonatal intensive care together with the challenges of caring for an extremely preterm infant in terms of both the emotional burden and impact on their family life. This input is ongoing. Our PPI coapplicants have both confirmed that the research question is very relevant and would make a meaningful difference to infants born prematurely and their long-term outlook. We also sought input from several parents (including those with preterm-born infants now aged around 1 year corrected) and asked them to outline the outcomes of most importance to parents of preterm-born babies. We have asked for their views on the design of the overall study, especially in terms of daily dosing requirement and practicalities as well as the optimal methods to collect data on a regular basis and to improve adherence. Since the use of the optimal primary outcome for respiratory disorders is fraught with controversy, we sought the views of ten additional parents and asked them to list the main outcomes they believe are most important to them from day/night-time wheezing, GP/A&E/Hospital admission, drug usage, longer term outcomes, etc. Most have agreed that visits to health professionals and long-term lung damage are to be avoided if at all possible. Thus, with the parents’ input, we revised the primary outcome to the one which we believe is the most important not only clinically but also to parents of this vulnerable group of infants.

Our PPI co-applicants are integral members on the Trial Management Group (TMG) to provide input on the day-to-day progress and management of the trial. They have had involvement in preparation of parent-facing materials and the parent App. This involved discussions of the time required from participants to complete the App. Two parent representatives are members of the Trial Steering committee (TSC).

Any dissemination material will be reviewed by our PPI coapplicants and the wider group as necessary.

### Trial procedures

#### Site selection and training

Site selection was/is based on receipt of an expression of interest and personal contact directly with the neonatal intensive care units. A registration questionnaire captured key information about the site, local site study team and any concerns around delivery of the protocol in the context of routine practice. A preliminary meeting was offered to the sites to present the study background and protocol. Formal site induction initiation visits were/are held either by remote video conferencing, in person at each site, or by hybrid of the two to train the local site study team in trial-specific procedures. Comprehensive guidance documents have also been produced. The local clinical trials pharmacy team is also provided with trial-specific training in IMP storage, accountability and reconciliation procedures during the initiation process. All local site study teams have undertaken Good Clinical Practice training commensurate with their roles and responsibilities.

### Participant recruitment

The total sample size is 542 infants randomised to receive Bactek sublingual spray or matching placebo (50:50 treatment allocation) up to 1 year of corrected age, to be administered by parents once daily (2× actuations of spray). Formal follow-up is being conducted by the research nurses at the recruiting sites at month 3, 6, 9 and 12 (1-year corrected age).

**Figure 1:**
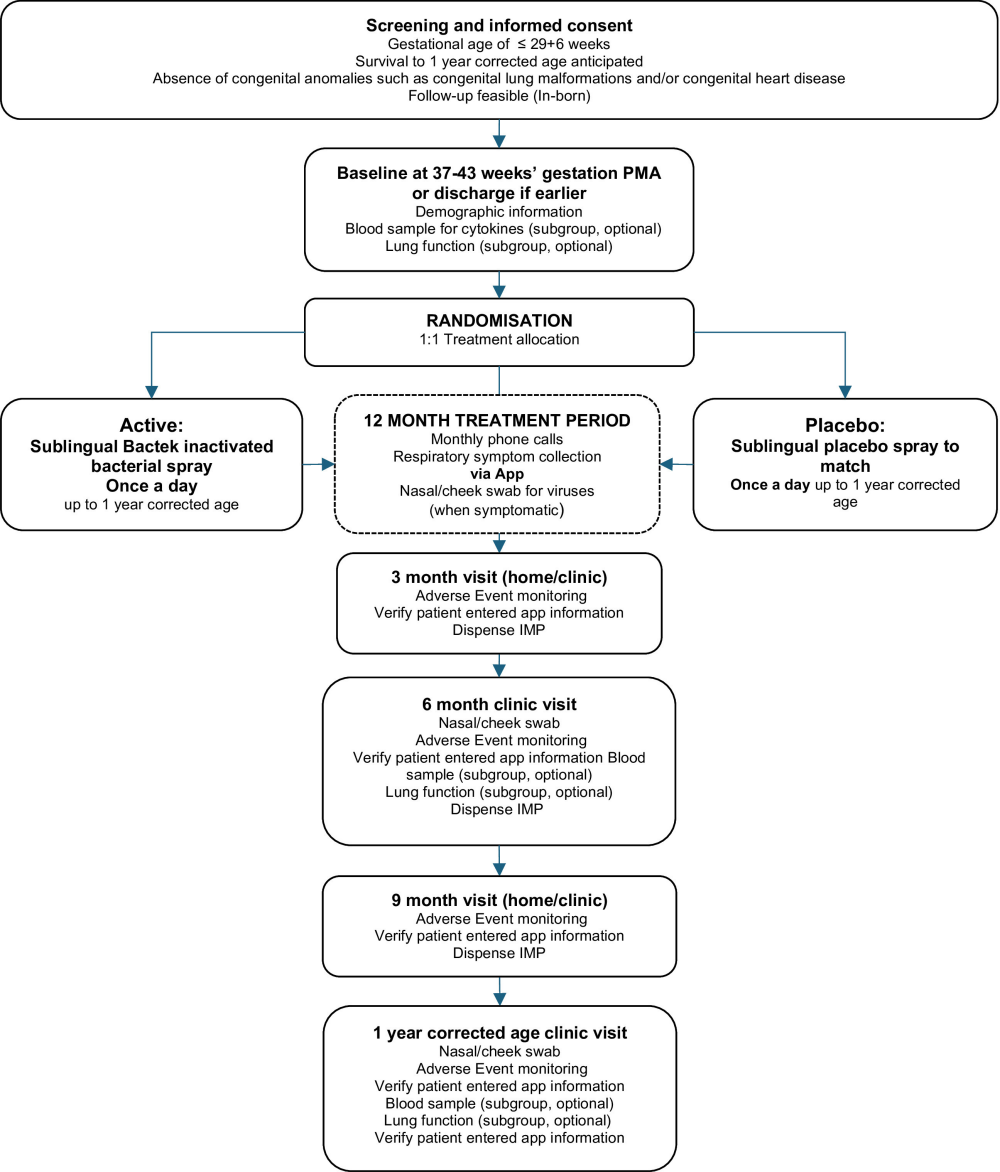
Participant flow diagram. IMP, Investigational Medicinal Product; PMA, postmenstrual age.

The trial design is summarised in figure 1. Recruitment is anticipated to take 24 months after a 3-month safety run-in period with an anticipated 12–18 sites participating.

### Screening and consent

All infants who meet the gestational age criteria for the trial are registered on an anonymous screening log. Reasons for not randomising otherwise eligible infants and reasons for excluding infants are recorded on the screening log.

The local site study team identifies potentially eligible preterm-born infants when the infants are either approaching 37 weeks’ PMA or prior to discharge if it is earlier. The parents receive a verbal description of the trial and if they are interested, the ethically approved parent/guardian information sheet and consent form are provided. Eligibility is confirmed by a medically qualified individual.

At sites participating in ancillary blood sample collection, the consent process includes an additional description of the procedures involved and plans to retain the collected samples for use in future research.

At sites participating in the ancillary lung function testing (impulse oscillometry), the consent process includes an additional description of the procedures involved.

Parents are given adequate time to read the information sheet and to ask questions. If the parents are willing to participate, they are asked to sign the consent form which is countersigned by a member of the local site study team. Maternal consent is required as maternal data is collected. Consent for future follow-up contact is optional. The right to withdraw from the study at any time without affecting their infant’s clinical care is clearly communicated. Please see the consent form in the online supplemental material.

### Randomisation

Once eligibility has been confirmed and the parents/guardians have consented, randomisation to the intervention is performed. The randomisation list was prepared by an independent CTR statistician. Participants are being randomised 1:1 using minimisation with a random element (p=0.8). Allocations are minimised by gestational age and anti-RSV prophylaxis—both assumed to have a strong prognostic relationship with the primary outcome. Maternal vaccination against RSV was introduced in the UK during the winter of 2024. Although universal anti-RSV prophylaxis may be introduced in the winter of 2025 to replace existing targeted anti-RSV treatment, we already have incorporated plans to stratify the intervention based on receipt of any anti-RSV prophylaxis. To avoid any issues with inadvertent unblinding or dosing errors, multiple births are being randomised to the same arm. Allocation to Bactek or placebo is double blinded, the allocation is not known to clinicians, the infant’s family or the study team with the exception of the independent CTR statistician. The list was uploaded to a web-based randomisation system provided by the Centre for Healthcare Randomised Trials (CHaRT) (University of Aberdeen). A member of staff at the recruiting site performs randomisation on the web-based randomisation system which produces the pack ID that the infant is allocated to and a participant ID. The BALLOON IMP is then prescribed by an appropriately qualified individual. If necessary, unblinding can be performed on the web-based randomisation system by the PI.

### Data collection and sampling

#### Clinical assessments

After documentation of eligibility and consent in the web-based randomisations system, subsequent data collection is in Research Electronic Data Capture (REDCap) by the recruiting site staff and parents/guardians complete an App provided by Your Research B.V. (Huizen, Netherlands) to record respiratory symptoms, non-respiratory symptoms and adherence to the IMP. A WheezeScan device is provided to all parent(s)/guardian(s) to detect and differentiate wheezing from other breath sounds. If respiratory symptoms are reported on the App, the recruiting site staff will be able to access the data including reporting of respiratory symptoms, as they will have access to the App dashboard to review data entries of all participants recruited from their site. They will then verify if an LRTI has occurred.

Initial data collection in REDCap focuses on maternal history, information about the family, antenatal information, details around the infant’s birth and neonatal admission. Monthly follow-up phone calls are being carried out by the site staff at 1, 2, 4, 5, 7, 8, 10 and 11 months of age focusing on adherence, concomitant medications and ensuring the parents have adequate BALLOON IMP and swabs for the next month and all LRTIs have been verified. Face-to-face visits either at the infant’s home or in hospital take place at 3, 6, 9 and 12 months of age. The face-to-face visits focus on IMP adherence, receipt of concomitant medications, supply of new IMP sprays and weighing the returned sprays (to further aid adherence), ensuring all LRTIs have been verified, and completing ongoing medical history including any potential adverse events.

Infants remain in follow-up for safety until 13 months’ corrected age.

### Data collection

All data will be recorded on the trial electronic Case Report Form in the CHaRT or REDCap systems. Data are regularly monitored and queries are raised as necessary with sites.

### Sampling

#### Viral swabs

If the infant has any respiratory symptoms, the App alerts the parents to obtain a viral swab with details of how to obtain one shown before discharge by the recruiting site staff. The App also alerts the recruiting site research nurse when a sample is obtained/required. Recruiting sites are provided with the required materials for sample collection, labelling and transport, which they give to the parents at each study visit. The parents will post the viral swabs directly to the Wales Specialist Virology Centre at Public Health Wales in Cardiff, for batch analysis for a broad range of common seasonal viruses. An educational video showing the parents how to obtain a viral swab is shown on the BALLOON public website.

### Analysis

#### Sample size

The primary analysis involves comparing health professional diagnosed LRTIs between enrolment and 1-year corrected age between the two trial arms. Based on rates of LRTIs from previous cohorts of preterm-born infants of 46%^3 4^ and the AZTEC follow-up data showing admission rates of 55% (of 385 survivors with available data) by 1 year of corrected age (unpublished data), we estimated that 55% of participants in the placebo arm would experience a health professional diagnosed LRTI.

Our target effect size is a 15% absolute reduction (ie, reduction to 40% in the intervention arm, translating to a relative difference of 27%). This is a realistic target and of clinical importance to clinicians, health providers and parents. We assume a loss to follow-up rate of 15%, as data will be obtained from healthcare professionals and followed up by research nurses and hence completion should be high.

In order to maximise trial efficiency and allow for stopping for lack of benefit, we have included an interim analysis (stage 1) as part of this trial with an intermediate outcome of health professional diagnosed LRTIs within 6 months post-randomisation. We assume a positive predictive value between intermediate and definitive primary outcome of 1 by design (given that the intermediate outcome is the definitive outcome measured at an earlier timepoint, and outcomes are cumulative) and that the majority (37%/55%) of LRTI diagnoses occur during the first 6 months post-randomisation.

To maintain overall power of 90% and a pairwise one-sided alpha of 0.025, we require 346 recruited participants, of whom 182 will have analysable intermediate outcome data, to conduct an interim analysis with stopping for lack of benefit. Our stage 1 one-sided alpha is 0.43 and power is 97%. The unblinded interim data will be assessed independently by the Independent Data Monitoring Committee (IDMC) and recommendations made to continue the trial or not.

Should we progress from the interim analyses to stage 2, we would require 542 total recruited participants of whom 460 have analysable definitive outcome data. The stage 2 one-sided alpha is 0.025 and power is 92%.

### Statistical analysis

#### Safety run-in period

Although Bactek has previously been used in preterm-born infants, we included a formalised safety run-in period as follows:

- Recruit and randomise the first four infants with the expectation that half of these participants would be allocated the active arm. The IDMC reviews the allocation and advises if more participants need to be recruited.
- Administer the study product as per protocol and monitor randomised infants for 1 week post-randomisation, ideally while still in hospital.
- Record any adverse events. If no adverse events are reported, write an email to the IDMC Chair declaring this.
- If there are adverse events, generate a safety report for IDMC and hold open and closed meetings to discuss the progression of the study.
- Decisions which the IDMC could make following the end of the safety run-in period:
  - Do not proceed with any further recruitment according to the current protocol and indefinitely pause the trial. This would be due to adverse events being reported which were deemed to be serious (defined as any untoward medical occurrence(s) that results in death, hospitalisation or prolongation of existing hospitalisation, persistent or significant disability/incapacity) and probably/definitely related to the study product. We would liaise with the local site study teams and manufacturer to determine the best way to resolve any issues that are identified.
  - IDMC to meet at 3 months to review safety on a greater number of participants. This would be due to either non-serious adverse events occurring which were probably or definitely related to study product or serious adverse events occurring which were possibly related to study product. Recruitment would continue during this period unless major safety concerns were identified by the TMG.
  - Continue recruitment for the rest of the internal pilot. This would be due to no adverse events occurring (confirmed via email to IDMC), non-serious adverse events which were at most possibly related, or serious adverse events which were at most unlikely related to the study product.
- IDMC writes a letter of recommendation to the TSC who in turn will write to the funder at the end of the safety run-in period.

During March to May 2025, four infants were recruited. There were no adverse events reported to the study team, which after assessment by the IDMC Chair, resulted in a decision by the TSC to commence full recruitment from June 2025.

### Interim analysis

At the interim analysis stage, we will compare the proportion of infants experiencing at least one health professional diagnosed LRTI within 6 months between trial arms by fitting a two-level Poisson (or negative binomial/logistic) regression model, with multiple infants nested within mothers and robust standard errors. Models will also adjust for gestational age and anti-RSV prophylaxis (as per randomisation factors). Results will be reported to our IDMC as adjusted risk differences (using the margins command in Stata), 95% CIs and p values. As per our design parameters, should our one-sided p value be >0.43, this would provide statistical indication of a lack of benefit of our intervention. Additional data (eg, other outcomes, including safety data) may be considered by our IDMC when deciding whether to progress or stop at this stage.

### Analysis of primary outcome

Analysis will be performed based on intention to treat. Our primary analysis will compare the proportion of participants receiving a health professional diagnosed LRTI post-randomisation between 37 and 43 weeks PMA or discharge if earlier and 1-year corrected age between arms by fitting a two-level Poisson (or negative binomial) regression model, with multiple births nested within mothers and robust SEs. Models will also adjust for gestational age and anti-RSV prophylaxis (as per randomisation factors). Results will be reported as risk ratios, 95% CIs and p values. Univariable, as well as adjusted models will be presented. Missing data are expected to be minimal but will be handled within a multiple imputation framework.

### Analysis of secondary outcome measures

Secondary outcomes will be analysed using appropriate regression models (eg, linear, Poisson, negative binomial, flexible parametric survival models), adjusting for stratification and minimisation variables as per the primary analysis.

### Subgroup analyses

We will extend our primary analysis by fitting trial arm by subgroup interaction terms to explore any differential intervention effects by gestational age and anti-RSV prophylaxis.

### Current status of trial

Recruitment started in March 2025 and the safety run-in completed in June 2025. Since no reported adverse events were reported, the IDMC gave the go-ahead to start full recruitment in June 2025. At the time of writing (25 September 2025), 31 infants have been recruited, and twelve recruiting sites were open with 3–4 due to open in the next 2 months.

## Ethics and dissemination

The current version of the BALLOON Protocol is 1.4, dated 4 June 2025. Ethics permission has been granted by the Wales Research Ethics Committee 3 (Ref 24/WA/0181), and regulatory permission by the Medicines and Healthcare Products Regulatory Agency (CTA reference 21323/0063/001-0004). NHS permission has been granted by the Health Research Authority (HRA) and capacity and capability confirmed by each individual NHS organisation. The study is registered on ISRCTN (ISRCTN14019493 https://doi.org/10.1186/ISRCTN14019493) and on the NIHR portfolio (CPMS 55712). Cardiff University is the sponsor (resgov@cardiff.ac.uk) and was not involved in the preparation of this manuscript or the decision to submit. Personal data is held with the explicit consent of participants, independently of study data. We shall disseminate our findings via national and international peer-reviewed journals and conferences. Oversight of the study is being performed by an IDMC (comprising two expert paediatricians and an expert statistician) and an independent TSC (comprising three expert paediatricians, an expert statistician and two lay representatives). Appointments to these committees were made with the approval of the NIHR and a meeting is being held at least annually.

## Discussion

Infants born preterm at ≤29+6 weeks’ gestation are at risk of lung disease in the early neonatal period and subsequently vulnerable to LRTIs during infancy and childhood. These LRTIs may lead to increased healthcare utilisation with visits to GPs, A&E and hospital admissions with time missed from work for the parents. In addition, the LRTIs may exacerbate the existing lung disease. Therefore, it is important to aim to prevent viral infections in infancy. Currently, preventative strategies are largely limited to anti-RSV antibody prophylaxis, maternal RSV immunisation during pregnancy and avoidance of viral infections especially in nurseries. TIBVs, such as Bactek, are increasingly used and provide a promising therapeutic avenue for preterm-born infants to target postdischarge respiratory viral infection in this vulnerable group of preterm-born infants.

We opted to study the preterm-born group below 30 weeks’ gestation at birth as this is the group at the highest risk of developing the important lung disease BPD in the neonatal period, which can have lifelong consequences and thus is an important group for intervention to prevent LRTIs after discharge from the neonatal unit. If this treatment is successful in decreasing LRTIs during the first year of life, there should be large cost savings for the health services throughout the world. The cost to the health service for each hospital admission for a chest infection is over £6000 (in 2012).^22^ Importantly, it will also benefit the parents and their infants. The definition of an LRTI may, in some cases, be difficult to discriminate from an upper RTI. However, any potential LRTI will be verified by research staff at the sites, so this should ensure that the primary outcome is accurate. We have also included the effects of LRTIs on the parents, including time off nurseries and off work. The results should be easily interpretable as we are adequately powered to meet the objectives of the study and will ensure adequate data are collected.

Throughout the development of the study, we have engaged parents who have been through the medical care of preterm-born babies at the extremes of prematurity, including those with BPD. Importantly, by surveying a significant number of parents in different UK neonatal centres, we were made aware of outcomes including LRTIs after discharge and long-term consequences of preterm delivery. We believe that with their input, we have developed an important study to address their important concerns of preventing hospitalisation and possibly long-term respiratory consequences of prematurity.

## Supplementary material

10.1136/bmjopen-2025-107929 online supplemental file 1
