# Supplementary material for: Randomised, double-blind, parallel group, placebo-controlled, trial of Bactek for the prevention of lower respiratory tract infections in preterm infants in the UK: BALLOON study – study protocol
Source: BMJ Open. 2026 Mar 9;16(3):e107929. doi: 10.1136/bmjopen-2025-107929 (PMC12983818; doi:10.1136/bmjopen-2025-107929)
Supplement: online supplemental file 1 [file bmjopen-16-3-s001.pdf]

Centre for Trials Research / Canolfan Ymchwil Treialon — Cardiff University / Prifysgol Caerdydd
University Hospitals of Leicester NHS Trust
The Newcastle upon Tyne Hospitals NHS Foundation Trust
Queen Mary University of London
FUNDED BY NIHR | National Institute for Health Research

<<NHS Organisation Logo>>
<PI name>
<Trust/Site address 1>
<Trust/Site address 2>
<Trust/Site address 3>
<postcode>
Tel: <telephone number>

BALLOON

Consent Form

Study ID: B A _ _ _ _ ***NHS/CHI NUMBER:*** _ _ _ _ _ _ _ _ _ _

To be completed by the Parent/Guardian

	Once you have read and understood each statement, please enter your initials in each box	Initial
1.	I have read and understood the information sheet for the BALLOON trial (Version 1.3, dated 20/06/2025). I have had the opportunity to ask questions and have had these answered satisfactorily.	initial
2.	I understand that my baby's participation is voluntary and that I am free to withdraw my baby from the study at any time, without giving a reason, and without my baby's care or legal rights being affected. I understand that in some cases further information about any unwanted effects of my baby's treatment may need to be collected by the study team.	initial
3.	I understand that, if I choose to withdraw, I have the option to have my baby's viral swabs destroyed. However, if it is not possible to ascertain my wishes at the point of withdrawal, I understand that the viral swabs will be used for the purposes of the trial as described in the information sheet.	initial
4.	I understand that my baby's data will be retained for a minimum of 25 years and that the data will be stored in a confidential manner.	initial
5.	I understand that information about both the mother and baby that is held and maintained by NHS England/ISD Scotland/NWIS/NDAU and other central UK NHS bodies, may be collected from medical records and other health-related records. These may be looked at by the research team and responsible practitioners during the trial. The Sponsor, NHS Organisation and Regulatory Authorities may also look at the data collected during the study. I give permission for these individuals to have access to these records and for them to be used in this research on the understanding that all information will remain confidential.	initial
6.	I understand that my GP will be contacted about my baby's participation, and that they may be contacted for updated information about their care, and the information used as part of the study. I understand that my GP may be contacted in the event the trial team has concerns about my child's wellbeing.	initial
7.	I understand that nose or cheek swab samples will be collected for this study. I understand that my baby's DNA/RNA will be extracted alongside the bacterial DNA/RNA but will not be used in the BALLOON study.	initial
8.	I agree to allow information or results arising from this study to be used in future healthcare and/or medical research in an anonymised form.	initial
9.	I give permission for a copy of my baby's consent form to be sent to the CTR at Cardiff University (where it will be kept in a secure location), so they can confirm that my consent was given.	initial
10.	I agree for my baby to take part in the above study.	initial

<<NHS Organisation Logo>>
<PI name>
<Trust/Site address 1>
<Trust/Site address 2>
<Trust/Site address 3>
<postcode>
Tel: <telephone number>

Consent Form

Study ID: | B | A | | | | | ***NHS/CHI NUMBER:*** | | | | | | | | | | |

Below are optional statements:

<<OPTIONAL STATEMENT FOR ALL CENTRES >>

11. I agree to gift any of my baby's remaining samples (including my baby's DNA/RNA) to be used in future prematurity research in the UK and abroad, which may include genetic (e.g. DNA) and commercial research. [initial]

12. I agree for my baby's follow up data on applicable databases (at this hospital, NHS digital/NHS Wales Informatics Service/ISD Scotland/NDAU) to be reviewed by Cardiff University researchers. [initial]

13. I agree for me and my baby's GP to be contacted by Cardiff University for the purposes of following up when my baby is two years of corrected age. I understand that contact details will be kept strictly confidential (according to the 2018 General Data Protection Regulation) and that no personal information will be used in study reports or publications. [initial]

14. I agree that I may be contacted in the future in relation to other research studies. [initial]

1. <<OPTIONAL STATEMENT FOR CENTRES IN ANCILLIARY STUDIES>>

2.

15. I agree for my baby to have their lung function measured during the study. [initial]

16. I agree for my baby to have blood samples taken as part of the study for use in exploratory research to find out more about how the Bactek works. [initial]

3. 17. I understand I am free to withdraw my consent for use of blood samples in exploratory research, and for future research at any point. However, if it is not possible to ascertain my wishes at the point of withdrawal, I consent to my baby's samples being used for the purposes as described in the information sheet. Please select YES/NO [initial]

4.

Baby's name (please print): ______________________________

Name of parent/guardian (please print): ______________________________ Your signature: __________________________

Relationship to baby: ____________________________ Date: ______________

<<NHS Organisation Logo>>
<PI name>
<Trust/Site address 1>
<Trust/Site address 2>
<Trust/Site address 3>
<postcode>
Tel: <telephone number>

Consent Form

Study ID: | B | A | | | | | ***NHS/CHI NUMBER:*** | | | | | | | | | | |

	2nd Parent name, (please print): ____________________________ Your signature: __________________________<br>Date: ______________<br><br>Researcher taking consent name (please print): _____________________________ Signature: ______________________________ Date: ______________

(1 copy for the participant, 1 copy for the study file, 1 copy for the medical notes)
