# Supplementary material for: Randomised, double-blind, parallel group, placebo-controlled, trial of Bactek for the prevention of lower respiratory tract infections in preterm infants in the UK: BALLOON study – study protocol
Source: BMJ Open. 2026 Mar 9;16(3):e107929. doi: 10.1136/bmjopen-2025-107929 (PMC12983818; doi:10.1136/bmjopen-2025-107929)
Supplement: Supplementary file 2 [file bmjopen-2025-107929.reviewer_comments.pdf]

PEER REVIEW HISTORY

BMJ Open publishes all reviews undertaken for accepted manuscripts. Reviewers are asked to complete a checklist review form and are provided with free text boxes to elaborate on their assessment. These free text comments are reproduced below.

ARTICLE DETAILS

Title (Provisional)

Study Protocol: A randomised, double-blind, parallel group, placebo controlled, trial of Bactek for the prevention of lower respiratory tract infections in preterm infants in the United Kingdom – BALLOON Study

Authors

Kotecha, Sarah Joanne; Lowe, John; Gillespie, David; Perez-Alijas, Mahuampi; Aboklaish, Ali; Mahachit, Tarirai Lincoln; Cumming, Oliver Sebastian; Harris, Debbie; Hubbard, Marie; Thomas-Jones, Emma; Jones, Tim; Ladell, Kirsten; Moore, Catherine; Humpreys, Iain; Grigg, Jonathan; Berrington, Janet; Kotecha, Sailesh

---

VERSION 1 - REVIEW

**Reviewer**	**1**
**Name**	**Tramper-Stranders, Gerdien**
**Affiliation**	**Franciscus Gasthuis en Vlietland, Pediatrics**
**Date**	**04-Sep-2025**
**COI**	**None**

This protocol paper by Kotecha et al. describes the study protocol for a trial using bacterial vaccines to prevent RTI in extremely preterm infants. This is a very important topic as RTI in infancy are a heavy burden for them and their parents.

The trial already started in 2025 and is currently recruiting, but not yet finished.

Below, I have written my comments.

General

In the introduction, a few concepts are missing. First, as “bacterial vaccines” are largely unknown, this might be explained better, including their working mechanism. The term “bacterial vaccine” might be misleading, as many colleagues may think of routine vaccinations; perhaps “immunostimulants” would be more precise. Second, cited studies only describe secondary prevention and mainly wheezing episodes. As the researchers aim to study primary prevention of respiratory tract infections, are there any data on this? They could expand a bit more on the possible differences (or in the discusion part).

Moreover, the referenced studies focus mainly on wheezing episodes, while the primary outcome here is LRTI. Are there any data on LRTI reduction after bacterial vaccine use?

Could the buren of respiratory disease in the first years after preterm birth a bit more explained? Why was this gestational age group chosen and not the full spectrum of PALD, as majority of preterm born infants are being born > 30 weeks of GA ?

The wheeze scan is very interesting but only mentioned in the abstract, should also be referred to in the methods.

Specific

Strengths Section:

Please consider:

Is there any proof that bacterial vaccines prevent lower RTI, the primary outcome?

How is lower RTI discriminated from viral wheezing?

Is there any evidence that it prevents lung damage? This could be more thoroughly described from a pathophysiological viewpoint.

Introduction:

Page 5, line 22: “preterm” is mentioned twice; this is redundant.

Page 5, line 33: “term born” is mentioned twice; the comma is redundant.

Lines 37–53: This paragraph could be written more concisely; for example, activation of innate and adaptive immunity is mentioned twice. Mechanistic work should be highlighted more: how is the vaccine administered (oral, sublingual), and what is its mode of action? How does it induce innate and adaptive responses? Role of PAMPS/TLR etc.

Page 5, line 60: What is a “respiratory exacerbation”?

Page 5, line 60: Were preterm-born children included? As far as I know, in the majority of studies this was an exclusion criterion.

Page 6, line 9: Please refer to evidence for decrease in Lower RTI.

Page 6, line 19: I am not sure whether this can be applied to the preterm population, as they were usually excluded or only included in very limited studies comprising a small proportion of participants.

Line 22: Could be more precise; namely, 3–5 is reported in the full cohort of Nieto, not in preterm infants, and those infants were on average 2 years old.

Page 6, line 24: “Only” is used twice in the sentence. Why is the focus on preterms <29.6 GA?

Page 6, line 35: Also, in infants of this age, e.g., <1 year old?

Methods:

I am not sure whether the methods section is following a specific format, but it feels somewhat disorganized and could be written more concisely, with some data moved to supplementary materials.

Page 6, lines 44–56: The objective is described both in the introduction and the methods.

Page 7, Design: Please mention the design here, not the sample size or allocation.

Population: Is there a minimum weight in the inclusion/exclusion criteria?

Page 9, Primary Outcome: This differs from what is reported in the abstract and objectives. Is it parent-reported, or only LRTI health care visits? Is it “AND” hospital admission, or “OR”?

Page 10: Fever and runny nose/moist cough are, in most infants, symptoms of an upper respiratory tract infection, as is poor feeding due to nasal congestion. Rethink the definition of lower RTI. It is often difficult for GP’s to discriminate (could be in discussion part).

What is the difference between the primary and first secondary outcome? Is the primary outcome the number of patients with one or more LRTI as parent-reported or doctor-diagnosed? It remains a bit unclear to me.

I am missing secondary outcomes on IOS measurements and biosampling, which are mentioned in the methods.

Page 11, line 25: Patient participation needs a separate paragraph, including how patients are involved in every phase of the study.

Page 11, line 55: Is RSV prophylaxis universal now in UK?

Page 15: In my opinion, the lung function training phase can be omitted or put in supplementary materials.

Page 16, Sample Size: Is there a rationale for the assumption of a 27% decrease based on earlier studies?

Page 18, Internal Politics and Progression Criteria: Might be transferred to supplementary data.

Page 19, line 13: Is there evidence that most LRTIs are expected in the first 6 months? I would think this is the other way around bt maybe there are some papers to proof.

Page 20, Sensitivity Analyses: I do not understand this sentence, and “we will consider” is not very strong.

Discussion:

The first two paragraphs of the discussion do not add new information.

Ethics and dissemination could be moved to the methods section.

I am missing a small discussion of the potential benefits for patients, interpretation, and foreseeable limitations, as indicated in the CONSORT checklist, also see my suggestions before.

Tables:

Tables 1 and 2 could be provided as supplementary information.

Good luck with the trial execution!

VERSION 1 - AUTHOR RESPONSE

Reviewer: 1
Dr. Gerdien Tramper-Stranders, Erasmus MC Sophia, Sint Franciscus Vlietland Groep
Comments to the Author:
This protocol paper by Kotecha et al. describes the study protocol for a trial using bacterial vaccines to prevent RTI in extremely preterm infants. This is a very important topic as RTI in infancy are a heavy burden for them and their parents.

The trial already started in 2025 and is currently recruiting, but not yet finished.

Below, I have written my comments.

Thank you for recognising the importance of the trial and your detailed review.

General
1. In the introduction, a few concepts are missing. First, as “bacterial vaccines” are largely unknown, this might be explained better, including their working mechanism. The term “bacterial vaccine” might be misleading, as many colleagues may think of routine vaccinations; perhaps “immunostimulants” would be more precise. Second, cited studies only describe secondary prevention and mainly wheezing episodes. As the researchers aim to study primary prevention of respiratory tract infections, are there any data on this? They could expand a bit more on the possible differences (or in the discussion part).

2. Moreover, the referenced studies focus mainly on wheezing episodes, while the primary outcome here is LRTI. Are there any data on LRTI reduction after bacterial vaccine use?

Thank you for this important comment. We have now refrained from using the term “bacterial vaccines” and changed this to the more accurate description of “trained immunity-based vaccines (TIBVs)” throughout the manuscript. We have also included the two references which were cited by Dr Tramper-Stranders in her recent protocol paper for this aspect (ref: 16 and ref: 17) as well other newer ones (ref:18 and ref:19).

3. Could the buren of respiratory disease in the first years after preterm birth a bit more explained? Why was this gestational age group chosen and not the full spectrum of PALD, as majority of preterm born infants are being born > 30 weeks of GA ?

The group of preterm born infants at highest risk of respiratory disease are those born at <30 weeks' gestation who are at most risk of developing the important disease process called Bronchopulmonary Dysplasia (BPD) with over 50% admitted during the first year of life for respiratory reasons, mainly LRTIs, thus we are full justified in studying this important group of infants at highest risk of PLD. We were already aware of Dr Tramper-Stranders' PROTEA study investigating a similar related product for later preterm born infants thus our study of the more at risk group is complimentary to PROTEA. We have, nevertheless, added this an explanation on page 22, second paragraph of the discussion).

4. The wheeze scan is very interesting but only mentioned in the abstract, should also be referred to in the methods.

We are pleased the reviewer found the use of WheezeScans interesting and have added it to the Methods section on Page 10, Line 15.

Specific

Strengths Section:
Please consider:

1. Is there any proof that bacterial vaccines prevent lower RTI, the primary outcome?

We have used a similar outcome of LRTI as Dr Tramper-Stranders' PROTEA study and used the same justifications for using LRTI as the primary outcome, which we are sure she will agree with! We have added more information about recent data showing prevention of LRTI by TIBV.

2. How is lower RTI discriminated from viral wheezing?

Please note that wheezing is a symptom and LRTI is a diagnosis thus if wheezing is noted then it is important to identify the underlying diagnosis as it can be due to a LRTI or it can be due to causes such as an underlying diagnosis of BPD, which many of these infants will have. It is for this reason that we had extensive discussion including with the grant body's Scientific Committee and their many Reviewers to extensively and accurately define LRTIs which we are confident we have done. We have added in the Methods section that the WheezeScan will be used to identify wheezing (as one of several symptoms).

3. Is there any evidence that it prevents lung damage? This could be more thoroughly described from a pathophysiological viewpoint.

A thorough description is beyond the scope of a protocol paper and would be better placed in a review paper (including *in vitro* and animal models, we are already considering such a review) – nevertheless, we have added to the Introduction (Page 4, line 10) to note evidence that preterm born infants who had viral infections had worse lung function at one year of corrected age when compared to those who did not.

Introduction:

1. Page 5, line 22: “preterm” is mentioned twice; this is redundant.

We have deleted one of the “preterm”.

2. Page 5, line 33: “term born” is mentioned twice; the comma is redundant.

We have revised this sentence.

3. Lines 37–53: This paragraph could be written more concisely; for example, activation of innate and adaptive immunity is mentioned twice. Mechanistic work should be highlighted more: how is the vaccine administered (oral, sublingual), and what is its mode of action? How does it induce innate and adaptive responses? Role of PAMPS/TLR etc.

We have revised the paragraph and deleted the duplicated phrase. We had already added the route of administration to the paragraph and referred the Reader to a manuscript detailing the mode of action as we believe it is beyond the remit of this manuscript to go into more detail in a protocol paper, which should focus on the clinical trial with sufficient *brief* background of the scientific rationale which we have covered. Additional mechanistic work including PAMPs and TLR/NLR, etc. should be covered in a specific review paper.

4. Page 5, line 60: What is a “respiratory exacerbation”?

Respiratory exacerbations are well described and well known to general practitioners, adult and paediatric respiratory e.g. for asthma, COPD, etc but we have added a line to explain them on page 5, line 5.

5. Page 5, line 60: Were preterm-born children included? As far as I know, in the majority of studies this was an exclusion criterion.

Many studies do not discriminate between term and preterm especially after 1-2 years of age, Indeed, 16.7% of the Nieto study included preterm born children which we have now clarified on page 5, line 23.

6. Page 6, line 9: Please refer to evidence for decrease in Lower RTI.

We have included data on the rates of respiratory tract infections after TIBVs and have also noted the lack of data for preterm-born infants in the introduction.

7. Page 6, line 19: I am not sure whether this can be applied to the preterm population, as they were usually excluded or only included in very limited studies comprising a small proportion of participants.

We have soften this sentence to “ likely to be applicable” rather than “are applicable”.

8. Line 22: Could be more precise; namely, 3–5 is reported in the full cohort of Nieto, not in preterm infants, and those infants were on average 2 years old.

We have revised the sentence so it states that the results were in the whole cohort of children including term born infants at a median age of 24 months and apologise for not doing so before.

9. Page 6, line 24: “Only” is used twice in the sentence. Why is the focus on preterms <29.6 GA?

Please note the two “only”s refer to two different aspects but we have altered the wording. We have removed one of the “only”. As above, the preterm group at the highest risk of developing BPD are those born at <30 weeks’ gestation, hence we are including those <30 weeks gestation which to avoid any confusion should be stated as 29 weeks and 6 days in the protocol.

10. Page 6, line 35: Also, in infants of this age, e.g., <1 year old?

We have clarified the age in this sentence.

Methods:
1. I am not sure whether the methods section is following a specific format, but it feels somewhat disorganized and could be written more concisely, with some data moved to supplementary materials.
We apologise that the reviewer found it disorganised but we have followed the recommended sections for a protocol paper. We would have benefitted from specific reasons and examples why the Reviewer feels the format is disorganised. Indeed, we believe that most are relevant in a protocol paper which should focus on the described elements for a clinical trial but would be happy to move any specific sections to the supplementary if they are specifically not relevant for a protocol paper.

2. Page 6, lines 44–56: The objective is described both in the introduction and the methods.

We have left the full objective in the methods and considerably shortened the objective in the introduction as we believe it is imperative to give a short objective in the introduction and then expand it in the methods.

3. Page 7, Design: Please mention the design here, not the sample size or allocation.

We have revised this section as requested and moved the sample size and allocation to elsewhere in the manuscript.

4. Population: Is there a minimum weight in the inclusion/exclusion criteria?

There are no specific weight criteria in the inclusion/exclusion criteria in the protocol hence it has not been included in the population section.

5. Page 9, Primary Outcome: This differs from what is reported in the abstract and objectives. Is it parent-reported, or only LRTI health care visits? Is it “AND” hospital admission, or “OR”?

We apologise for the confusion - the LRTI will first be reported by the parents on the App and then verified by the research staff in the sites and should be “OR”. We have revised this section in the manuscript.

6. Page 10: Fever and runny nose/moist cough are, in most infants, symptoms of an upper respiratory tract infection, as is poor feeding due to nasal congestion. Rethink the definition of lower RTI. It is often difficult for GP’s to discriminate (could be in discussion part).

Absolutely correct but many apologies for the reviewer’s misinterpretation as it should be noted that these symptoms are ***alerts*** to identify a ***potential*** URTI/LRTI and also note that ***additional*** symptoms, as described, are required to alert health professionals for a potential diagnosis of LRTI. A specific LRTI verification form will diagnose LRTI as confirmed by a health professional (or not) as described. We have had extensive discussions with world experts and also reviewed many protocols including PROTEA & others, and believe our definition is far more accurate and prescriptive than those in other clinical trials which are vague asking any health professional to diagnose LRTI with no guidance thus resulting in great variation for diagnosing LRTIs between health professionals. We do not believe we need to make any changes and would challenge those just adding “Health Professional (or Doctor) Diagnosed LRTIs or wheezing” as a primary as being appropriate.

7. What is the difference between the primary and first secondary outcome? Is the primary outcome the number of patients with one or more LRTI as parent-reported or doctor-diagnosed? It remains a bit unclear to me.
It is incredibly important to accurately describe the LRTI as there are many options as clearly described in our outcomes. Number of LRTIs is inappropriate as a primary outcome as the data is skewed towards only on 0-1 for each child thus yes/no is more appropriate as we have opted. We have clarified this more in the manuscript (Page 10, line 4).

8. I am missing secondary outcomes on IOS measurements and biosampling, which are mentioned in the methods.

Blood analyses and lung function data will be reported separately from the main trial as is usually for such studies. As a result we have minimised their mention in the paper. We have clarified this on page 7, line 3.

9. Page 11, line 25: Patient participation needs a separate paragraph, including how patients are involved in every phase of the study.

We have included a separate paragraph in the participant recruitment which details how many patients are involved in the main trial. We have also added a PPI section as requested by the Editor.

10. Page 11, line 55: Is RSV prophylaxis universal now in UK?

Maternal RSV immunisation from 28 weeks was introduced in winter 2024 (with variable uptake) and nirsevimab is likely to be introduced in winter 2025 although many will receive palivizumab as at present if the former is not available. We therefore, already have minimisation by RSV prophylaxis as mentioned in the methods. We had added this information into the manuscript (on page 13, line 23).

11. Page 15: In my opinion, the lung function training phase can be omitted or put in supplementary materials.

We have minimised these sections and removed the training aspect as we agree is less relevant.

12. Page 16, Sample Size: Is there a rationale for the assumption of a 27% decrease based on earlier studies?

The absolute improvement of 15% is based on clinically important outcome and feasible to achieve with the intervention. Any higher is unlikely to be achieved by the intervention and any lower is unlikely to be clinically important. We obviously had such discussions at length locally and with the funders and their peer reviewers and are very satisfied with our overall study design.

13. Page 18, Internal Politics and Progression Criteria: Might be transferred to supplementary data.

We have removed these.

14. Page 19, line 13: Is there evidence that most LRTIs are expected in the first 6 months? I would think this is the other way around bt maybe there are some papers to proof.

We were a little uncertain of this comment esp. as there is ample evidence that LRTIs are greatest during the first year of life in extremely preterm after discharge but the Reviewer says its “the other way around”, can she please explain why she thinks this? It may be that she counts the early days after preterm birth at <30 weeks’ gestation (prior to term corrected age) when infections are low during neonatal stay but generally when studies report that infections are greatest during the first year if life, this is after discharge to home (around term equivalent) esp as many of these infants are discharged home on oxygen and thus have low respiratory reserve thus are compromised even with “minor” infections. The greatest risk of admission is during the first 3-6 months of life hence anti-RSV administration occurs during this time period. We have added a reference for high rates of LRTI during the first year of life but would

welcome any additional explanation of the Reviewer's for stating "the other way around".

15. Page 20, Sensitivity Analyses: I do not understand this sentence, and "we will consider" is not very strong.

As is usual, sensitivity analyses will be developed as part of the statistical analyses plan thus we have removed this section.

Discussion:

1.The first two paragraphs of the discussion do not add new information.

We have revised the two paragraphs but do need to give a summary of the aims.

2. Ethics and dissemination could be moved to the methods section.

We have moved this section to the Methods section.

3. I am missing a small discussion of the potential benefits for patients, interpretation, and foreseeable limitations, as indicated in the CONSORT checklist, also see my suggestions before.

The Editor has asked us to replace the CONSORT checklist with the SPIRIT checklist. However, we have added in the requested information to the discussion.

Tables:

1. Tables 1 and 2 could be provided as supplementary information.

We have removed both the tables.

VERSION 2 - REVIEW

**Reviewer**	**1**
**Name**	**Tramper-Stranders, Gerdien**
**Affiliation**	**Franciscus Gasthuis en Vlietland, Pediatrics**
**Date**	**13-Oct-2025**
**COI**	

Dear authors,

I want to thank you for addressing my questions thoroughly and adjusting the manuscript. In my opinion, it has largely improved. I have some minor comments.

Strengths and Limitations session

* Points 1 and 4 appear to overlap in content and could therefore be merged for conciseness and clarity.

Introduction

* The genus name S. epidermidis should be written in full, just as the other bacterial names.

* I don't agree with the statement that "these studies have often included significant numbers of preterm-born infants/children"; in fact, most have not included large numbers of preterm-born participants, as also acknowledged in the strengths and limitations section.

* The sentence "Thus, there is growing proof of concept that Bactek provides a promising therapeutic avenue as was noted in a recent review [19]" describes an opinion article, not a review. Could be omitted, this comes from data which is already referred to.

*Consider focusing on one name of bacterial immunostimulant (e.g., MV130) rather than discussing both MV130 and Bactek for consistency (eg. just as OM-85 is mentioned and not Broncho-Vaxom)

* In the following sentences, there is a redundant use of "however":

"However, there is supporting evidence including Nieto's MV130 study [8] reporting fewer wheezing attacks (3.0 vs 5.0) in the whole cohort (from which 16.7% were preterm-born children) at a median age of 24 months.. However,"

* "We are aware of another trial that is ongoing…" sounds conversational. Consider rephrasing to a more scientific tone, such as:

"An additional ongoing trial is investigating OM-85 in infants born after 30–36 weeks of gestation, which will broaden the evidence base across the spectrum of prematurity."

Methods

* Great to see a section on Patient and Public Involvement (PPI), it could be a bit condensed ; the current level of detail may be more appropriate for supplementary material.

* In Participant recruitment, the word "ages" appears redundant and can be omitted.

* Response Point 14: I understand your evidence and agree that most severe infections often occur at very young age, such as RSV. I was mainly thinking about a term age group in which the frequency of RTI increases after ~6 months because of waning passive immunity (breast feeding, maternal IgG) and starting with day care attendance. Most viral encounters are after 6 months of age (https://pmc.ncbi.nlm.nih.gov/articles/PMC7107408/pdf/jix599.pdf), including RTI with wheezing, although preterm infants represent a distinct group.

*Page 16: The statistical plan includes the description of the interim analysis. I am sorry to bring this up now, but I do not completely understand the sentence 'Our Stage 1 one-sided alpha is 0.43 and power is 97%...' and the power calculation for the interim analysis (only

N=182 needed for analysis at 6 months? Or 346?). As I noticed I was the only reviewer for this manuscript, might be reviewed by an expert as I do not feel myself experienced enough in this part.

*The detailed description of the safety run-in period may be excessive for the main methods section. Consider summarizing the key aspects and relocating the extended description to the supplementary materials.

Best regards,

**Reviewer**	**2**
**Name**	**Martin, James**
**Affiliation**	**University of Birmingham, Institute of Applied Health Research**
**Date**	**25-Nov-2025**
**COI**	

Thank you for submitted this re-submitted protocol. I have some minor comments before I can recommend publications.

I feel the sentence: "They are being randomised to receive Bactek or placebo, once daily dose of 2 sprays (each 0.1ml) of IMP (300 (Formazin Turbidity Units) FTU), from 37-43 weeks' PMA or discharge if earlier up to 1 year of corrected age." In the abstract could be written in a clearer manner. For example, I am not sure at this point whether the daily dose of 2 sprays is part of the Bactek group, the placebo group, or both groups. Here, you also mention about recruiting from 37-43 weeks PMA or discharge up to 1 year. This is also presented in the introduction section of the abstract. I don't think you need it in both places. I would arguably keep in in the methods section – though perhaps as a separate sentence, rather than linked to the sentence on randomisation.

In the abstract, you use LRTI without first introducing the acronym.

In the abstract, you introduce the primary outcome as health professional diagnosed LRTIs, and the first secondary outcome as health-professional confirmed unscheduled visits for LRTIs. What is the difference between these outcomes – as it is not clear to me at first.

For any multiple births, are you including all of the children from the birth in the study? If so, how will you acknowledge this in the analysis?

The second inclusion criteria is about whether follow-up is feasible. How is this going to be defined and captured in a way that doesn't make the study at risk of recruitment bias. For example, can the PI selectively exclude patients (who may be allocated to the placebo or Bactek) in a manner which may impact the results? Linked to this, how are you going to ensure blinding and allocation concealment?

In the abstract, the primary outcome is defined as: "The primary objective is to assess if sublingual Bactek spray, decreases the risk of health professional diagnosed LRTIs (unscheduled visits to general practitioners (GPs), accident and emergency departments (A&Es) and hospital admissions) between enrolment and one year of corrected age. Secondary outcomes include: the number of parent-reported, health professional-confirmed unscheduled visits for LRTIs…' In the methods section of the main manuscript, the primary outcome is defined as: "The primary outcome is the presence/absence of parent-reported, health professional-confirmed unscheduled visits for LRTIs". Perhaps I am misunderstanding, but it looks to me as though the primary outcome in your main body of text is listed as a secondary outcome in the abstract. Note, in your trial registry, you use: "the risk of health professional diagnosed LRTIs". Can you clearly articulate what the primary outcome is, and ensure the language is consistent across documents. Equally, can you clearly define how this is being measured. The use of "risk" and "presence/absence" would lead me to believe this is a binary outcome – whereby the presence of any one of these components would lead to a positive for the primary outcome. Is this correct? Is there a rationale for using a binary composite outcome and not a count outcome? Similarly, it would be useful to comment on the appropriateness of combining these to form a single outcome.

You are planning an interim analysis. What approaches are you taking to maintain the overall type 1 error rate?

In the statistical analysis section, you report a section on subgroup analysis before the section on the primary analysis. The sub-group analysis section should come at the end of the statistical analysis section.

In the interim analysis stage, you plan to estimate risk differences only. At the final analysis stage, you plan to report risk ratios only. Is there a reason for reporting differently at each stage? Additionally, CONSORT recommend publishing both absolute and relative effect measures, and so I would recommend publishing both risk ratios and risk differences at the end of the trial.

In the "analysis of primary outcome" section, the analysis is described as: "Univariable, as well as adjusted models will be presented.". For the univariable analysis, is this an unadjusted analysis? In the adjusted models, is this the models that include gestational age and anti-RSV prophylaxis, or are there additional covariates that will be included. Given that you should be including the randomisation factors in the analysis, its not clear what the rationale is for an unadjusted analysis. Further, I would like it to be clearer that the adjusted analysis is the primary analysis.

In the analysis of secondary outcome measures, it is stated: "Secondary outcomes will be analysed using appropriate regression models (e.g., linear, Poisson, negative binomial, flexible parametric survival models), adjusting for stratification and minimisation variables as per the primary analysis." What are the stratification variables that are being adjusted for here?

Can I clarify whether the second secondary outcome (time to first parent-reported unscheduled visit) is modelled as time-to-event? Are you planning on using Poisson or Cox regression models for this? And how will you deal with patients that don't have a first unscheduled visit.

VERSION 2 - AUTHOR RESPONSE

Reviewer: 1
Dr. Gerdien Tramper-Stranders, Franciscus Gasthuis en Vlietland
Comments to the Author:

1. I want to thank you for addressing my questions thoroughly and adjusting the manuscript. In my opinion, it has largely improved. I have some minor comments.
Thank you, we are pleased you think the manuscript has improved.

2. Strengths and Limitations session
   * Points 1 and 4 appear to overlap in content and could therefore be merged for conciseness and clarity.

We have merged points 1 and 4 for clarity and conciseness.

Introduction
3. The genus name S. epidermidis should be written in full, just as the other bacterial names.
We have given this in full now and thank the reviewer for pointing out the omission.

4. I don't agree with the statement that "these studies have often included significant numbers of preterm-born infants/children"; in fact, most have not included large numbers of preterm-born participants, as also acknowledged in the strengths and limitations section.
Whilst we do not agree with the reviewer's comment (e.g. Nieto's study stated 15% of their population was born preterm which is high and significant esp when compared to rates of 7-8% rates of preterm births in Europe), we have revised this statement to now say a "small number" rather than "significant numbers".

5. The sentence "Thus, there is growing proof of concept that Bactek provides a promising therapeutic avenue as was noted in a recent review [19]" describes an opinion article, not a review. Could be omitted, this comes from data which is already referred to.
Again we believe the Review synthesises evidence of Bactek showing therapeutic promise in our population, we have deleted the reference to the review.

6. Consider focusing on one name of bacterial immunostimulant (e.g., MV130) rather than discussing both MV130 and Bactek for consistency (eg. just as OM-85 is mentioned and not Broncho-Vaxom)

Many thanks for this comment we have ensured that we refer to Bactek throughout the paper but have left MV130 in twice, once in the abstract and once in the introduction so that readers are aware of the relationship between the names.

7. In the following sentences, there is a redundant use of “however”:
“However, there is supporting evidence including Nieto’s MV130 study [8] reporting fewer wheezing attacks (3.0 vs 5.0) in the whole cohort (from which 16.7% were preterm-born children) at a median age of 24 months.. However,”
We have deleted the second “However”.

8. “We are aware of another trial that is ongoing...” sounds conversational. Consider rephrasing to a more scientific tone, such as:
“An additional ongoing trial is investigating OM-85 in infants born after 30–36 weeks of gestation, which will broaden the evidence base across the spectrum of prematurity.”
We have revised the sentence as suggested by the Reviewer and thank them for the comment.

Methods
9.Great to see a section on Patient and Public Involvement (PPI), it could be a bit condensed ; the current level of detail may be more appropriate for supplementary material.
As PPI input is essential and crucial to every stage of development of the modern clinical trial, we would like to leave this important section in as it is. However, if the Editor believes that the PPI input is less relevant then we would be happy to move some of the PPI content to the online supplement.

10. In Participant recruitment, the word “ages” appears redundant and can be omitted.
We have deleted the redundant ages from that section.

11. Response Point 14: I understand your evidence and agree that most severe infections often occur at very young age, such as RSV. I was mainly thinking about a term age group in which the frequency of RTI increases after ~6 months because of waning passive immunity (breast feeding, maternal IgG) and starting with day care attendance. Most viral encounters are after 6 months of age (https://pmc.ncbi.nlm.nih.gov/articles/PMC7107408/pdf/jix599.pdf), including RTI with wheezing, although preterm infants represent a distinct group.
Thank you for the clarification. As this trial is recruiting very preterm born infants we would prefer to leave the statement as it is but thank the reviewer for their help with this.

12. Page 16: The statistical plan includes the description of the interim analysis. I am sorry to bring this up now, but I do not completely understand the sentence ‘Our Stage 1 one-sided alpha is 0.43 and power is 97%...' and the power calculation for the interim analysis (only N=182 needed for analysis at 6 months? Or 346?). As I

noticed I was the only reviewer for this manuscript, might be reviewed by an expert as I do not feel myself experienced enough in this part.
As discussed above, the detailed statistical analyses plan (SAP) is usually developed during the recruitment phase with expert input from our statistical team, TSC and IDMC. We shall aim to publish the SAP separately as we did for a previous study (ref: Lowe et al, Trials 2022;23(1):704). We believe it is inappropriate to give extensive details in this publication esp as the contents may change with expert input from the trial team, TSC and IDMC. As stated above, we would be happy to discuss this important aspect with the Editor.

13. The detailed description of the safety run-in period may be excessive for the main methods section. Consider summarizing the key aspects and relocating the extended description to the supplementary materials.
We believe the safety run in period is critical to the safe running of the trial and would like to leave the detail in this section so it is clear to the readers. However, if the editor would prefer we would be happy to move some of the content to the online supplement.

Reviewer: 2
Dr. James Martin, University of Birmingham
Comments to the Author:
Thank you for submitted this re-submitted protocol. I have some minor comments before I can recommend publications.

I feel the sentence: "They are being randomised to receive Bactek or placebo, once daily dose of 2 sprays (each 0.1ml) of IMP (300 (Formazin Turbidity Units) FTU), from 37-43 weeks' PMA or discharge if earlier up to 1 year of corrected age." In the abstract could be written in a clearer manner. For example, I am not sure at this point whether the daily dose of 2 sprays is part of the Bactek group, the placebo group, or both groups. Here, you also mention about recruiting from 37-43 weeks PMA or discharge up to 1 year. This is also presented in the introduction section of the abstract. I don't think you need it in both places. I would arguably keep in in the methods section – though perhaps as a separate sentence, rather than linked to the sentence on randomisation.

Since it states, "They are being randomised to receive Bactek or placebo, once daily dose of 2 sprays (each 0.1ml) of IMP (300, Formazin Turbidity Units, FTU), from 37-43 weeks' PMA or discharge if earlier up to 1 year of corrected age.", we believe that it is clear that the same "once daily dose of 2 sprays" is given.

In the abstract, you use LRTI without first introducing the acronym.

This has been corrected.

In the abstract, you introduce the primary outcome as health professional diagnosed LRTIs, and the first secondary outcome as health-professional confirmed

unscheduled visits for LRTIs. What is the difference between these outcomes – as it is not clear to me at first.

The difference on careful reading is that the primary outcome is *Yes/No* for LRTIs and the secondary is *number* of LRTIs.

For any multiple births, are you including all of the children from the birth in the study? If so, how will you acknowledge this in the analysis?

If parents agree, yes for all multiples and this will be acknowledged in the analyses.

The second inclusion criteria is about whether follow-up is feasible. How is this going to be defined and captured in a way that doesn't make the study at risk of recruitment bias. For example, can the PI selectively exclude patients (who may be allocated to the placebo or Bactek) in a manner which may impact the results? Linked to this, how are you going to ensure blinding and allocation concealment?

This is a pragmatic criterion used locally to ensure follow up is possible. The local PIs will be able to ascertain this. This is quite standard for such studies.

In the abstract, the primary outcome is defined as: "The primary objective is to assess if sublingual Bactek spray, decreases the risk of health professional diagnosed LRTIs (unscheduled visits to general practitioners (GPs), accident and emergency departments (A&Es) and hospital admissions) between enrolment and one year of corrected age. Secondary outcomes include: the number of parent-reported, health professional-confirmed unscheduled visits for LRTIs…' In the methods section of the main manuscript, the primary outcome is defined as: "The primary outcome is the presence/absence of parent-reported, health professional-confirmed unscheduled visits for LRTIs". Perhaps I am misunderstanding, but it looks to me as though the primary outcome in your main body of text is listed as a secondary outcome in the abstract. Note, in your trial registry, you use: "the risk of health professional diagnosed LRTIs". Can you clearly articulate what the primary outcome is, and ensure the language is consistent across documents. Equally, can you clearly define how this is being measured. The use of "risk" and "presence/absence" would lead me to believe this is a binary outcome – whereby the presence of any one of these components would lead to a positive for the primary outcome. Is this correct? Is there a rationale for using a binary composite outcome and not a count outcome? Similarly, it would be useful to comment on the appropriateness of combining these to form a single outcome.

We have thought this through extensively including with the funding committee and believe our approach is correct (optimal) esp as numbers are heavily skewed.

You are planning an interim analysis. What approaches are you taking to maintain the overall type 1 error rate?

This will and comments below will be covered in more detailed SAP which is being developed during the recruitment phase and will submitted for publication as with previous trials of ours (Lowe et al, Trials 2022;23(1):704).

In the statistical analysis section, you report a section on subgroup analysis before the section on the primary analysis. The sub-group analysis section should come at the end of the statistical analysis section.

In the interim analysis stage, you plan to estimate risk differences only. At the final analysis stage, you plan to report risk ratios only. Is there a reason for reporting differently at each stage? Additionally, CONSORT recommend publishing both absolute and relative effect measures, and so I would recommend publishing both risk ratios and risk differences at the end of the trial.

In the "analysis of primary outcome" section, the analysis is described as: "Univariable, as well as adjusted models will be presented.". For the univariable analysis, is this an unadjusted analysis? In the adjusted models, is this the models that include gestational age and anti-RSV prophylaxis, or are there additional covariates that will be included. Given that you should be including the randomisation factors in the analysis, its not clear what the rationale is for an unadjusted analysis. Further, I would like it to be clearer that the adjusted analysis is the primary analysis.

In the analysis of secondary outcome measures, it is stated: "Secondary outcomes will be analysed using appropriate regression models (e.g., linear, Poisson, negative binomial, flexible parametric survival models), adjusting for stratification and minimisation variables as per the primary analysis." What are the stratification variables that are being adjusted for here?

Can I clarify whether the second secondary outcome (time to first parent-reported unscheduled visit) is modelled as time-to-event? Are you planning on using Poisson or Cox regression models for this? And how will you deal with patients that don't have a first unscheduled visit.

We believe that we have addressed all the Editor's and Dr Tramper-Stranders' comments and addressed the relevant statistical reviewer's comments. Those relevant to a SAP will be included in the final SAP which is currently being developed. We strongly believe that these would be inappropriate in a protocol paper (few protocol papers including in BMJ Open publish detailed SAPs) as may change during the detailed SAP development period thus would be inaccurate if included in this manuscript. We believe we will include all the relevant comments anyway but are grateful for the reviewer's detailed comments.

VERSION 3 - REVIEW

Reviewer	1

Name Tramper-Stranders, Gerdien

Affiliation Franciscus Gasthuis en Vlietland, Pediatrics

Date 03-Feb-2026

COI

Most of my questions have been addressed, except for those raised by myself and the other reviewer regarding the (interim) analysis. While I agree that a detailed statistical analysis plan is typically developed during the study and is not required at this stage, we have provided several comments to improve the clarity and order of the summary of the planned statistical analysis in the current manuscript, which have not been addressed.

I leave it to the editor and other reviewer whether this should be changed.

VERSION 3 - AUTHOR RESPONSE

Reviewer: 1
Dr. Gerdien Tramper-Stranders, Franciscus Gasthuis en Vlietland
Comments to the Author:
Most of my questions have been addressed, except for those raised by myself and the other reviewer regarding the (interim) analysis. While I agree that a detailed statistical analysis plan is typically developed during the study and is not required at this stage, we have provided several comments to improve the clarity and order of the summary of the planned statistical analysis in the current manuscript, which have not been addressed.

I leave it to the editor and other reviewer whether this should be changed.

We are pleased that Dr Tamper agrees with us that the SAP is developed during the recruitment phase of the study. We have ensured her comment about "ordering statistical analyses" is now appropriate.
